# Developmental and Evolutionary Heart Adaptations Through Structure–Function Relationships

**DOI:** 10.3390/jcdd12030083

**Published:** 2025-02-22

**Authors:** Makena Phillips, Marina Nimmo, Sandra Rugonyi

**Affiliations:** Biomedical Engineering Department, Oregon Health & Science University, Portland, OR 97239, USA; phillmak@ohsu.edu (M.P.); nimmom@ohsu.edu (M.N.)

**Keywords:** hemodynamics, congenital heart disease, cardiac adaptation, mechanotransduction, environmental effects

## Abstract

While the heart works as an efficient pump, it also has a high level of adaptivity by changing its structure to maintain function during healthy and diseased states. In this Review, we present examples of structure–function relationships across species and throughout embryonic development in mammals and birds. We also summarize current research on avian models aiming at understanding how biophysical and biological mechanisms closely interact during heart formation. We conclude by underscoring similarities between cardiac adaptations and structural changes over developmental and evolutionary time scales and how understanding the mechanisms behind these adaptations can help prevent or alleviate the effects of cardiac malformations and contribute to cardiac regeneration efforts.

## 1. Introduction

Throughout evolution, species have adapted to diverse environments and demands. The structure of the heart has also changed, leading to various cardiac shapes and biophysical mechanisms to move blood through the body [1,2,3,4,5,6]. In evolution and embryonic development, both genetics and biophysics play a large role in determining the heart structure [7,8,9,10,11,12,13,14,15,16,17]. During the evolution of multicellular life, natural selection led to the development of diverse mechanisms that allow organisms to adapt to the environment. These include mechano-sensing and mechano-transduction mechanisms, which today continue to play an essential role in species survival [18,19,20,21,22,23,24,25,26,27]. These mechanisms, which appeared in response to evolutionary pressures, also helped advance cardiac structure and function over evolutionary time as well as ensure continuous fine-tuning of cardiac structure and function in an organism’s life. During the lifetime, the heart adapts in a number of ways to keep functioning even in suboptimal conditions (e.g., low oxygen, abnormal blood flow, over-/under-nutrition), but these same adaptative mechanisms can lead to congenital heart disease during development and to cardiac dysfunction and heart failure later in life [28,29,30,31,32,33].

In this Review, we present some of the evolutionary structural heart adaptations that appear in different species and examples of how species hearts function. We also discuss how the avian and mammal hearts adapt during embryonic development to changing metabolic needs and how the developing heart responds to biophysical cues. We highlight parallelisms between evolutionary and developmental cardiac changes that occur over drastically different timescales. In the last part of this review, we focus on avian models and summarize current research to understand how biophysical and biological factors closely interact during heart formation. We conclude by stressing the tight interaction between cardiac structure and function, pointing to the need for future research to uncover complex interactions between biological and biophysical factors that shaped the heart during evolution and continue to shape the heart during development and through remodeling, and could aid in cardiac regeneration efforts.

## 2. Heart Evolution

The first appearance of contractile proteins, which eventually allow cells to contract, occurred early in the evolution of multicellular life, most likely during the Paleoproterozoic Era, around 2 billion years ago [34,35,36]. Over evolutionary time, contractile cells organized into primitive tubes [35,37,38] and developed peristaltic-like contraction patterns that forced fluid through the tube, initially with no optimization for unidirectional flow [3,4,6,37,39,40]. As evolution progressed, bodily systems began to separate and become enclosed, such as in the phylum Chordata, allowing the emergence of closed circulatory systems [41,42]. In fact, the chordate’s primitive tubular pumping structure can be deemed as the ‘blueprint’ for cardiac circulatory systems in both invertebrates and vertebrates through conserved homologies [4,6].

**Figure 1:**
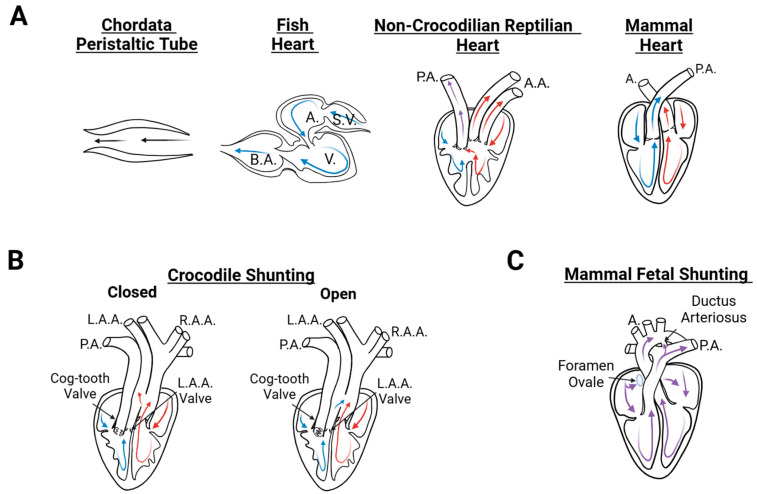
Examples of heart anatomy in different species reflecting evolutionary changes and adaptations. (**A**) Diverse cardiac morphologies starting with the primitive chordata peristaltic tube, considered the blueprint of cardiac circulation; the fish heart with separate chambers and valves in between chambers to facilitate unidirectional flow; the non-crocodilian reptile heart that exhibits partial ventricular septation and thus partial separation of pulmonary and systemic circulations and a heavily trabeculated myocardium indicated by wiggly wall lines; and the four-chambered heart in mammals, which allows complete separation of pulmonary and systemic circulations. Note that birds also have four-chambered hearts but with thicker walls than mammals. (**B**) Crocodile heart. The four chambers allow complete separation of pulmonary and systemic circulation. The ventricular myocardium is also heavily trabeculated (more than in mammals). Shunting allows crocodile blood flow to bypass the pulmonary circulation (when the pulmonary cog-tooth valve is closed) and diverts right ventricular blood flow to the systemic circulation through the left aortic arch when diving. (**C**) Fetal mammal heart. When fully formed, the fetal heart has four chambers and physical shunts, the foramen ovale that connects the two atria and the ductus arteriosus that redirects blood from the pulmonary to the systemic circulation. Shunting allows blood flow to bypass the pulmonary circulation until the fetus is born. Red arrows denote oxygen-rich blood, blue arrows denote oxygen-poor blood, and purple denotes blood oxygenated through the placenta. A.: atrium; V.: ventricle; S.V.: sinus venosus; B.A.: bulbus arteriosus; P.A.: pulmonary artery; A.: aorta; A.A.: aortic arches; L.A.A. and R.A.A.: left and right aortic arches, respectively. Created with BioRender.com: https://www.biorender.com/ (accessed on 14 February 2025).

The primitive fluid-pumping tube soon progressed from the chordates to vertebrates, creating a starting point for cardiac formation that later led to more complicated cardiac anatomies (see Figure 1A). The evolution of heart structures as critical features of circulatory systems across different species is highly debated [2,3,38,43,44,45,46,47,48,49]. Structural heart changes that were advantageous appeared in response to evolutionary pressures and through structural tinkering [50]. Meanwhile, the cardiovascular system continued to evolve to satisfy the growing demands of diverse species, such as performance, metabolism, and independence from surroundings [3,51]. Diverse factors were at play for each cardiac feature that appeared over evolutionary time, with many hypothesized to develop convergently to similar structures [1,47,52]. Today, the vertebrate cardiovascular system varies significantly among species, with some exhibiting a single circulatory system (e.g., in fish) and others two separate circulatory systems (e.g., in mammals and birds) to accommodate air-breathing and higher metabolic demands. The heart structure, in turn, also differs to allow single or parallel circulations.

The teleost heart has four separate chambers that are connected in series. From inlet to outlet, these chambers are the sinus venosus, atrium, ventricle, and bulbus arteriosus (see Figure 1A). Unlike in primitive tubular structures, the cardiac chambers are separated by valves that open to allow forward blood flow and close to block blood from flowing backwards [53], supporting unidirectional flow [1]. The teleost heart contracts from inflow to outflow, taking in deoxygenated blood and pumping it to the gills for oxygenation [54], from where blood is directed to the rest of the body [1]. This single circulation system is enough to satisfy the fish metabolic demands.

As life forms made their way onto land, air-breathing vertebrates emerged, and with them, the need to circulate blood to the lungs. Circulatory systems are separated into two parallel systems: (i) a systemic circulation that supplies blood to the body and (ii) a pulmonary circulation that allows blood flow through the lungs. Unlike fish and amphibians, reptiles only get oxygen from the lungs [55,56,57,58,59,60]. The non-crocodilian reptile heart has two separate atria but only one ventricle with partial separation (septation) and a heavy sponge-like trabeculation [43,52,61,62,63] (see Figure 1A). This ventricle acts as a pressure pump that sends blood to multiple outlets, but blood can mix within the ventricle, leading to partially separated pulmonary and systemic circulations [64]. Reptiles, however, have two aortic ventricular outlets and the ability to shunt blood so that it either flows through the lungs or bypasses the lungs depending on need (e.g., air-breathing vs. diving, respectively) [55,56,57,58,59,60]. Shunting allows for blood to be directed through different paths [49,63], presenting a structure–function relationship [49,65] that has been hypothesized as a ‘physiological benefit’ in the reptilian species [55,66].

The crocodilian heart, like avian and mammalian hearts, has two fully separated ventricles: a right and a left ventricle [2,52,63]. Yet, like other reptiles, crocodiles have two aortic outlets (see Figure 1B) and possess the ability to shunt blood flow depending on activity (e.g., air-breathing vs. diving) [7,52,67,68]. In contrast, adult avian and mammalian hearts, like the human heart, also have two ventricles but only one aortic outlet and no shunting [44,52,69]. Full ventricular septation allows complete separation of oxygenated and deoxygenated blood [3], as well as efficient motion of blood, with higher left ventricular blood pressure that enables circulation through the whole body and lower right ventricular blood pressure to sustain pulmonary circulation [3,70]. The separation of circulations allows for higher oxygen demands through more efficient oxygen transport [3,43,71].

In diving species, other adaptations, such as distinct hemoglobin and myoglobin affinity for oxygen and the ability to lower heart rate, are also important [72]. Reptiles have lower metabolic needs than mammals and birds. Reptilian hemoglobin, which carries oxygen in red blood cells, and reptilian myoglobin, an oxygen-binding protein in muscle, have lower oxygen affinity than their mammalian counterparts [73,74,75]. Upon diving, oxygen storage in reptiles remains mainly in the lung gas [72]. While a lower oxygen affinity reduces oxygen uptake and saturation, it also facilitates the release of oxygen to tissue cells. In reptiles, long dives are supported by the oxygen in their large (with respect to body ratio) lungs [76]. Hemoglobin and myoglobin affinities are also affected by environmental factors and activities (e.g., pH, temperature, partial oxygen pressure). In leatherback sea turtles, for example, oxygen affinity (and delivery) is controlled by pressure changes during diving that, together with lower heart rates, allow longer dives [77,78]. Crocodiles can stay submerged for longer periods of time thanks to their blood-shunting mechanisms and dropping of hemoglobin affinity that allows the release of oxygen to cells [79,80,81]. Diving mammals and birds have higher hemoglobin and myoglobin affinity, and thus more oxygen available in blood and stored in their muscles [72,82,83] to sustain their higher metabolic demands during diving. Their heart rates, moreover, can slow drastically during diving (bradycardia), reducing metabolic needs [84]. In king penguins, for example, large oxygen storage in blood and muscle, together with lower temperature and bradycardia during diving, enable longer dive times [85]. Among mammals, whales have the greatest dive depths and durations [86,87]. Whales swim slower than other aquatic mammals [86] and experience bradycardia, allowing them to preserve oxygen levels [88]. In addition, whales have higher myoglobin affinity for oxygen [89] that enables them to store larger amounts of oxygen in muscle tissue, a reserve that enables oxygen supply to tissues during long dives.

Interestingly, diving birds and mammals, unlike crocodiles, do not have physical vascular shunts to bypass the lungs during diving, but instead have large capacitance vasculature that allows them to change vascular resistance (e.g., increase pulmonary vascular resistance), enabling blood shunting during diving [84,90]. Reptilians, as well as diving birds and mammals, therefore, differ in their cardiovascular structure and response to fulfill the need to shunt blood during diving.

Together with changes in cardiac structure, the heart conduction system also evolved and today differs among species. An excellent review of evolutionary and developmental changes in the cardiac conduction system and how it differs among species has been recently published [91]. The cardiac conduction system initiates and coordinates electrical signals that synchronize cardiac contraction [92]. In the zebrafish heart, a single sinoatrial node generates contraction at the sinus venosus–atrium transition [93]. The signals then travel through cardiac trabeculations and compact myocardium to the atrio-ventricular canal and then the ventricle [91,93]. In mammals, birds, and crocodiles, two nodes are present: the sinoatrial node that acts as the heart pacemaker and sends signals to the atria, and an atrio-ventricular node that allows impulses to travel to the ventricles and is located at the basal portion of the interventricular septum [91]. Unlike in the zebrafish heart, in these four chamber hearts with separate circulations, atria and ventricles are electrically insulated from each other: impulses are sent from the sinoatrial node to the atria and the atrioventricular node and from there to the ventricles [94,95]. Interestingly, in non-crocodile reptiles, the atria and partially septated ventricle are electrically connected, allowing impulses to freely travel from the sinoatrial node to the ventricular myocardial cells [91]. Like other heart adaptations, an increasingly sophisticated organization of the conduction system has allowed species to sustain increased metabolic needs and demands.

Regardless of species, the heart’s structure directly impacts its function, and functional needs over evolutionary time scales have generally led to structural changes [2,55,56,63,66,70,96,97,98], together with other adaptations (e.g., changes in hemoglobin and myoglobin amounts and affinities in vertebrates). Significant cardiac structural changes also occur during heart development as the embryo grows from a single cell to an enclosed body with organs and circulation.

## 3. Heart Development

During mammal and avian embryonic development, the heart undergoes a transformation from a simple tubular structure with a single circulation to a four-chamber heart with systemic and pulmonary circulations [99,100,101]. This happens as the embryo grows and metabolic demands increase, culminating with birth or hatching, when the systemic and pulmonary circulations separate [102,103,104]. Moreover, in developing birds and mammals, shunting occurs during the embryonic and fetal developmental stages (see Figure 1C) to bypass the developing lungs [105,106,107]. Embryonic development, in a way, can be considered a prime example of an adaptation to metabolic and functional needs in an individual. This adaptation continues, although at a slower pace, throughout the lifespan [108,109,110].

**Figure 2:**
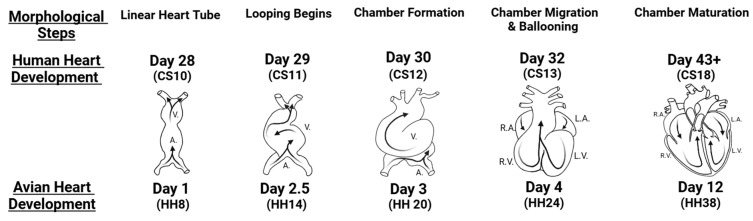
Heart developmental stages. Birds and humans (and mammals in general) have very similar heart developmental processes. Human development (upper labels) occurs over 9 months, with morphological heart characteristics appearing roughly on the specified days post fertilization and Carnegie stages (CS). Chicken development (lower labels) occurs over 21 days of incubation, with morphological heart characteristics occurring at approximately indicated incubation days and Hamburger–Hamilton (HH) developmental stages. A.: primitive atrium; V.: primitive ventricle; R.A. and L.A.: right and left atrium, respectively; R.V. and L.V.: right and left ventricle, respectively. Created with BioRender.com: https://www.biorender.com/ (accessed on 28 January 2025).

Mammalian cardiac development (see Figure 2) begins when the cardiogenic mesoderm folds [47,99,111,112,113], creating a linear heart tube [47,114,115]. Evolutionarily, this folding process becomes a key step in vertebrate heart development [47,113,116,117] and becomes an essential moment in embryogenesis [4,118]. This initial tubular heart consists of two cell layers: an inner endocardial cell layer and an outer contractile myocardial cell layer with an extracellular matrix (ECM) layer sandwiched in between [99,119]. The tubular heart functions like the primitive chordate tube, with the myocardium generating a peristaltic-like contraction pattern that pushes blood through the heart and into the circulation [47,95,120,121,122], initially with flow in both directions and progressively becoming unidirectional flow [123]. Soon after the heart tube is formed, endocardial cushions, which are thickenings of the cardiac wall ECM, develop in the heart atrioventricular canal and outflow tract [99,124]. The endocardial cells lining the cushions then undergo an endocardial-mesenchymal transition that populates the cushion ECM with mesenchymal cells [125,126,127,128]. Early during development, endocardial cushions act as primitive valves, limiting back flow upon myocardial contraction and promoting the forward movement of the blood [112,119,129,130]. Endocardial cushions also separate the heart into three consecutive sections: the primitive atrium, the primitive ventricle, and the outflow tract [112,115,122,131,132,133,134,135]. Meanwhile, upon heart tube formation, the proliferation of cells from the first heart field ceases, and the second heart field cells proliferate and then migrate into the heart, causing the heart tube to lengthen and start looping [99,136,137]. As the tube loops, the middle section bends out to the right laterally, creating a c-shaped heart that continues to loop into an s-shaped heart [119,131,136,138]. Within the heart tube, second heart field cells differentiate into myocardial cells. Neural crest cells migrate into the heart through the distal part of the outflow tract [139,140]. Second heart field and neural crest cells are needed for cardiac septation as the heart continues to develop [128,139,141,142].

Cardiac septation molds the developing heart into a four-chamber heart [44,143]. The outflow tract separates into the pulmonary artery and aorta, with endocardial cushions in the outflow tract giving rise to the aorticopulmonary septum as well as semilunar valves in the aorta and pulmonary artery [112,140,144,145]. Atrioventricular cushions fuse and divide the atrioventricular canal [112], leading to the formation of mitral and tricuspid valves [128,146,147]. Moreover, septal walls grow in the primitive atrium and primitive ventricle and eventually divide into left and right atrial and ventricular chambers, respectively [112,114,117,146,148,149]. Hence, during development, and while blood flow is already circulating, the ventricle becomes partially septated, similar to the non-crocodilian reptile heart (compare Figure 1A and Figure 2). To support continuous beating, the conduction system also adapts during cardiac development [91,92]. Like in zebrafish hearts, in the tubular heart, contraction is initiated at the sinoatrial portion of the heart, and impulses then travel to the ventricle. Later during development, the sinoatrial node is incorporated into the atria and becomes the heart pacemaker, while atria and ventricles isolate electrically during septation [91]. After ventricular septation, the atrio-ventricular node transmits signals to the ventricular myocardium through the septum. These changes in the conduction system occur while blood is flowing through the heart and are known to be affected by hemodynamics [16,91,150].

Once the heart is fully formed, before birth, a perforation in the septum between the two atria, known as the foramen ovale, connects the atria [117,151,152], and the ductus arteriosus connects the pulmonary artery to the aorta [65,103,152]. Both the foramen ovale and ductus arteriosus are temporary shunts with similar functions to the shunts found in crocodilian hearts, as they allow blood to bypass the pulmonary circulation during fetal stages [103,153]. Oxygenated blood from the placenta enters the heart through the right atrium and can flow through the foramen ovale to the left atrium (bypassing the lungs) and from there to the left ventricle [153]. Additionally, the ductus arteriosus redirects blood flow in the pulmonary artery to the aorta so that it bypasses the pulmonary circulation [2,103,153,154]. Fetal hemoglobin, moreover, binds to oxygen with higher affinity than adult hemoglobin, allowing the fetus’s blood to become oxygenated from the maternal circulation [155]. These shunts close soon after birth as the lungs become functional. This closure is essential to separate the systemic and pulmonary circulations [103,104,117,156].

Avian cardiac embryonic development follows a similar pattern to mammalian heart development [97,112,115,133,134,143,157,158]; hence, it is frequently used as a model of human cardiac development (Figure 2) [69,101,112,143,159]. A different staging system is employed for humans, other mammals, and chickens, but morphologically the hearts are similar [160,161,162]. There are some differences, however, including the chicken right atrioventricular valve is a muscular flap, while the human right atrioventricular valve (the tricuspid valve) has three leaflets; the chicken left atrioventricular valve is tricuspid, while the human left atrioventricular valve (the mitral valve) is bicuspid [112]. Moreover, to sustain flying, the avian heart has thicker walls and is bigger (per body mass) and, therefore, is more ‘muscular’ (as the increased mass is mainly due to additional muscle mass) than the mammalian, including human, heart [69,112].

Throughout evolution and development, the heart has changed its form and function to efficiently move blood through the circulation with minimal energy expenditure [52,163,164,165,166,167] and to quickly adapt to body demands (e.g., beating faster under physical exertion) [168].

## 4. Cardiac Mechanobiology: Evolutionary Traits and Malformations

Multicellular evolutionary and developmental processes rely on cell sensitivity to the bio-mechanical environment in which they are immersed. Mechanical stimuli on cells (stresses and stretches) elicit biochemical responses, which depend on the stimuli itself, the cell type, connections to neighboring cells, and the cell surrounding ECM. In turn, these mechano-transduction responses modulate gene expression patterns and genetic programming. Mechano-transduction mechanisms have developed over evolutionary timescales and are highly conserved among diverse species, especially vertebrates [169]. In fact, the ability to adapt to the mechanics of the environment is key both for evolution and embryonic development, as organs (including the heart) change in response to diverse stimuli, increasing chances of survival. Evolutionary and developmental adaptations include changes to both form and function that allow species to thrive in diverse environments.

Within the cardiovascular system, the movement of blood before, during, and after the development of the four-chambered heart—and its associated vasculature—imposes mechanical stresses on cardiovascular walls and valve tissues, which are also subjected to cyclic tissue stretch and compression [30,170,171,172]. It is well known that heart development is mediated by genetic programming but strongly regulated by environmental factors, including blood flow dynamics, which provide essential stimuli to heart cells. Hemodynamic stresses, especially blood pressure and wall shear stress, play a critical role in cardiac mechanical function and morphological development [150,173,174,175,176,177] and in cardiovascular tissue remodeling over the animal’s lifetime [172,178,179].

### 4.1. Cardiac Pumping and Flow Mechanics

In humans, like in birds and mammals, the four cardiac chambers utilize valves and muscle contractions to control blood flow into and out of the heart [180,181]. Because blood flows into relatively large cardiac chambers, the flow profile does not remain strictly laminar: flow jets and flow separation from walls lead to a swirling motion of the blood and vortex formation [165]. Indeed, within the heart, blood flows more efficiently in a swirling, vortical pattern than in a straight-line pattern [165]. As blood passes through the mitral valve opening into the much larger LV during diastole, vortex rings form, grow, and transport blood through the LV over the initial and late diastolic filling phases, promoting efficient flow through the ventricle [165,182,183,184]. Meanwhile, smaller vortices that form behind the leaflets of the mitral valve aid in valve closure when the flow of blood into the ventricle slows [183,184,185]. Following the closure of the mitral valve, contraction of the LV will force blood out of the ventricle and into the aorta by opening the aortic valve [186].

The cyclic contraction and expansion of cardiac walls generate pulsatile blood flow and regulate cardiac blood pressure. Cardiac cells respond to the biophysical stresses imposed by the flowing blood, as well as strains (cell deformations) they experience, modulating cardiac tissue growth and remodeling. Over time, mechanical stimuli exerted on cardiac cells (stresses and strains) modulate heart form and function and serve as a feedback loop for the growing heart to satisfy mechanical and metabolic demands.

### 4.2. Cardiac Mechanotransduction

The heart consists of different cell types. Endothelial cells are in direct contact with blood flow and myocardial cells, the heart muscle cells, represent most cardiac cells. Hemodynamic stresses on endothelial cells modulate cell morphology, growth, and the production and secretion of ECM components [178,187,188,189]. Endothelial cells are sensitive to the wall shear stress (friction force per unit area) exerted by flowing blood [150,187,190,191]. Transcriptional regulation in response to wall shear stress occurs through several mechanisms, including shear stress response elements, shear stress receptors, and the cell cytoskeleton [10,189,192,193,194]. Shear stress response elements are promoter sequences that mediate transcriptional responses to flow-induced shear stress [150,174,175,176,193,194,195]. Shear stress receptors act upstream of transcriptional factors and activators of heart morphology gene regulation, generating signaling cascades in response to wall shear stress [192]. Meanwhile, the cell cytoskeleton deforms in response to hemodynamic stresses, including shear stress, and transmits forces and stresses throughout the cell, regulating cell morphology and the force transmitted to the cell nucleus [196]. Cadherins form an adhesion complex that physically connects the cell cytoskeleton to neighboring cells [197,198,199]. Focal adhesion junctions involve integrin molecules physically attaching the cytoskeleton to the surrounding ECM [199]. Together, focal adhesions and focal adhesion receptors contribute to mechanically induced signaling and transcription [200], while mechanical stresses also regulate focal adhesion assembly [201,202]. Cytoskeletal attachments to neighboring cells and the ECM facilitate biomechanical coupling among cells and the tissues in which they are immersed, as well as signaling in response to mechanical stimuli (stresses and strains/stretches) [197].

Cardiac cells are also sensitive to cyclic stretching, resulting from contraction/relaxation cycles [203,204,205,206]. In addition to the cell cytoskeleton, which deforms with the cyclic stretching/contraction (signaling in response to cyclic deformation), stretch-activated ion channels contribute to cell signaling and mechanotransduction responses in cardiac cells [200,207,208,209,210,211,212]. Within cardiomyocytes, contractile filaments arrange together in an array to form myofibrils, which run parallel to the myocardial cell longitudinal axis [202,213]. Z-disks are observed at both ends of myofibrils and serve as a link between the myofibrils, ECM, and neighboring myocardial cells [214,215,216]. Intermediate filaments connect the inner Z-disk to the nucleus and focal adhesion junctions [201,206]. This complex but highly organized arrangement of molecules within cardiomyocytes not only serves to orchestrate cell contraction and relaxation but is highly involved in stress and stretch-induced signaling, and thus the response of myocardial cells to their biomechanical environment [203,205,213,214,216,217,218].

Overall, cardiac cells are highly sensitive to changes in mechanical signals, both originating from the interior of the cell and the environment [212,219,220]. For these signaling pathways to work, there needs to be communication from the nucleus to the cell’s environment. This is accomplished, along with other complexes, by the Linker of Nucleoskeleton and Cytoskeleton (LINC) complex, which provides structural stability to the nucleus while connecting the cytoskeleton to the nucleus [221,222,223]. LINC allows mechanically induced signals to impact transcription factor activity [223,224]. Thus, environmental factors, including hemodynamically induced stresses and stretches, modulate genetic programs. Disruptions in normal regulation patterns (e.g., due to genetic anomalies or abnormal hemodynamics) lead to abnormal cell function and alterations in the heart’s form, with a wide array of malformations leading to abnormal heart function.

**Figure 3:**
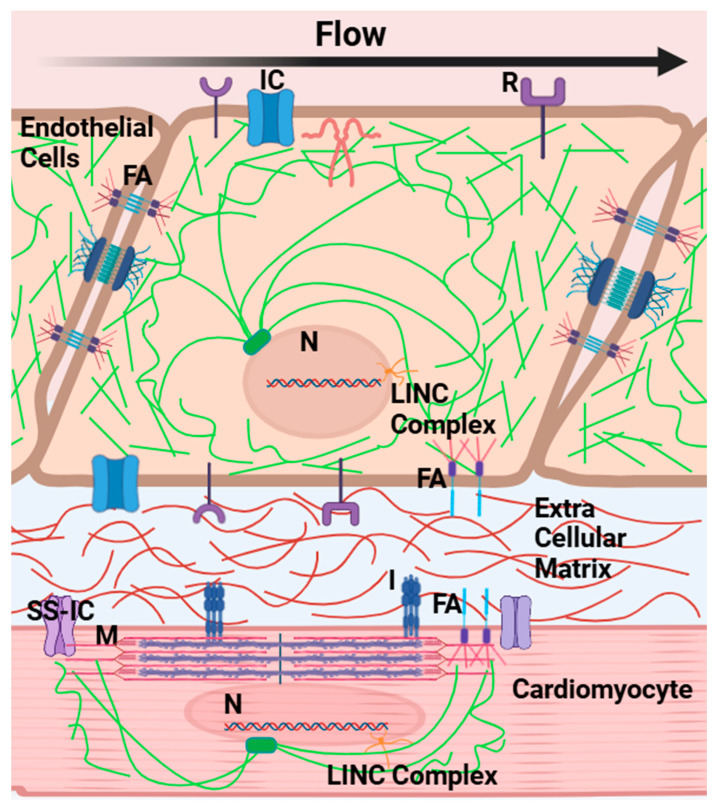
Sketch of cellular mechano-sensing and mechano-transduction mechanisms. Endothelial cells (**top**) and myocardial cells (**bottom**) are equipped with mechanisms that allow them to sense mechanical stimuli, such as stresses and stretches. In the heart, cells are particularly sensitive to hemodynamic stresses (including wall shear stress and blood pressure) and the cyclic contraction and relaxation of heart tissues. To sense and respond to hemodynamic stimuli, cells have stress-sensitive ion channels and receptors, as well as focal adhesion, cell–cell and cell–ECM complexes that connect the cell membrane to the cytoskeleton (green), the ECM environment (red in between cells) and neighboring cells, so that mechanical stimuli is transmitted throughout the cell and to the nucleus for transcription. Many proteins within cardiac cells and the cardiac cell contraction units, the myofibrils, are also sensitive to mechanics. A complex network of mechano-sensing and mechano-transduction signaling ensures that cardiac cells sense and respond to blood flow. In fact, both genetics and hemodynamic forces play a crucial role in heart formation as well as heart function later in life. R: receptor; I: Integrin; IC: ion channel; SS-IC: stretch sensitive ion channel; FA: focal adhesion; M: myofibril; N: nucleus. Created with BioRender.com: https://www.biorender.com/ (accessed on 28 January 2025).

While responses to hemodynamic-induced stresses and strains occur through several linked mechanotransduction mechanisms (see Figure 3), the net result is cardiac adaptation to the mechanical environment [30,172,174,187,212,224]. Notwithstanding the actual mechanism of action, a few simple rules have been successfully applied in the cardiovascular field to predict structural changes in response to physiological perturbations. First, increases in blood pressure lead to increases in wall thickness. Second, increases in blood flow velocity, and thus wall shear stress, lead to increases in vessel diameter [179,219,225,226]. From an engineering perspective, a simple concept applies to the examples above: changes occur to maintain homeostatic tissue stresses [174,227].

### 4.3. Cardiac Maladaptation and Congenital Heart Defects

To increase chances of survival during embryonic development, the heart adapts by changing its structure and/or its mechanical function in response to suboptimal conditions (e.g., abnormal pressure, blood flow velocities, oxygenation) [228,229]. Adaptations to abnormal environments, however, can also result in heart defects.

Congenital heart disease has a multifactorial etiology, with both chromosomal (genetic) anomalies and environmental insults playing critical roles in heart formation [174,230,231,232,233]. Factors responsible for the formation of heart defects include abnormal blood flow mechanics or anomalies in genes that sense or respond to hemodynamics [234].

**Figure 4:**
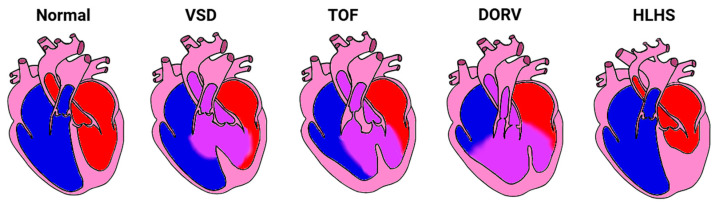
Sketch of the normal heart and hearts with common congenital heart defects. A normal heart (left) followed by depictions of cardiac malformations (from left to right): ventricular septal defect (VSD), tetralogy of Fallot (TOF), double outlet right ventricle (DORV), and hypoplastic left heart syndrome (HLHS). Figure was created based on the human heart, but mammal and avian hearts can reproduce these defects. Blue indicates deoxygenated blood, red indicates oxygenated blood and purple mixed blood. Created with BioRender.com: https://www.biorender.com/ (accessed on 16 February 2025).

The heart changes in response to abnormal blood flow, even at the early stages of embryonic development [174,176,235]. Embryonic blood flow can be perturbed by diverse environmental factors, such as toxins, placental anomalies, and maternal health [236,237]. Some heart defects that result are considered simple, e.g., hypertrophic cardiomyopathy (HCM), ventricular septal defect (VSD), while others are considered complex, e.g., tetralogy of Fallot (TOF), double outlet right ventricle (DORV), and hypoplastic left heart syndrome (HLHS) [170,238,239,240]. In humans, these heart defects (Figure 4) are associated with diverse genetic anomalies, which explain some but not all cases, e.g., [234,241,242,243,244]. In animal models, these heart defects can be induced by altering hemodynamics early during development, e.g., [234,235,245,246]. This suggests that both genetics and hemodynamics play fundamental roles in heart formation.

In HCM, LV walls are thicker than normal and, hence, stiffer [228,247,248]. The increased wall thickness can lead to a reduction in the volume of the LV lumen, affecting hemodynamics throughout the cardiac cycle [249]. The combination of reduced ventricular lumen and wall elasticity decreases ventricular filling during diastole due to limited LV chamber expansion and can impact the volume of blood ejected during systole (stroke volume) and the blood volume flow rate [8,250,251]. In about two-thirds of HCM patients, a thicker LV septum partially obstructs the ventricular outflow, increasing peak systolic blood pressure, which then leads to LV walls thickening further to sustain the higher pressure [252]. This could lead to a vicious cycle of deteriorating suboptimal conditions to which the heart continuously attempts to adapt to maintain function, but that can eventually lead to heart failure [228,253,254,255].

VSD is the most common congenital heart malformation [256] and is characterized by a lack of complete septation between the ventricles [238,257]. A VSD leads to the mixing of blood and changes in flow patterns, especially during systole, due to the movement of blood from the higher-pressure LV to the lower-pressure RV [28,258]. In many cases, smaller VSDs repair on their own without intervention [259]. Large VSDs, on the other hand, may lead to an increase in pulmonary blood pressure and congestion and thus may require surgical repair [259,260]. In more complex cases of congenital heart disease, VSDs are combined with other cardiac malformations (see below) [257].

TOF affects three of every 10,000 live human births, accounting for 7–10% of all congenital heart malformations [261]. Infants from mothers with untreated diabetes, phenylketonuria, or intake of retinoic acid are at an increased risk, and it is often accompanied by chromosomal trisomy [261]. About 10% of TOF cases are due to 22q11.2 deletion syndrome (also called DiGeorge syndrome), the most common TOF-associated genetic variant that can be inherited. Other genetic variants (e.g., trisomy 21 or Down syndrome and 45, X0 or Turner syndrome) have also been associated with TOF, which is considered a multifactorial heart disease [262,263]. TOF is characterized by four cardinal features, including pulmonary atresia or stenosis and a VSD, which vary in severity, affecting the manifestation and management of the disease [264]. In TOF, due to the pulmonary artery obstruction, flow is redirected to the aorta through the VSD. Increased flow through the aorta and diminished flow through the pulmonary artery can then affect aortic and pulmonary valve development and function during fetal stages [265,266]. Following diagnosis by echocardiography, surgical intervention and medical management at an early age significantly decreases morbidity and mortality in TOF patients [267,268]. Chronic complications include pulmonary regurgitation, recurrence of pulmonary stenosis, and ventricular arrhythmias [261].

DORV accounts for 1–3% of diagnosed congenital heart malformations in newborns [266,269]. It is diagnosed most frequently in fetuses with other anomalies, which leads to high intrauterine and postnatal loss due to terminations or declined postnatal therapy [270,271]. Anomalies such as trisomy 13 or 18 or 22q11.2 deletion syndrome put embryos at higher risk of developing DORV [271]. The presentation of DORV involves the connection of both great arteries either completely or predominately towards the RV and a VSD [272,273]. DORV can occur with transposition of the great arteries or without it. With no additional anomalies, infants have a good prognosis, but about 60% of cases may end up with single ventricle palliation [271], a condition in which only one functional ventricle remains to pump blood.

HLHS is caused by obstruction of flow into the LV during development. The reduction in flow through the LV inhibits the growth of the ventricle [274]. While HLHS accounts for about 1.4–3.8% of all congenital heart disease incidence, it is responsible for 23% of all cardiac deaths in the first week of life [275]. In infants with HLHS, the LV is severely underdeveloped, practically resulting in a single ventricle heart defect [276]. Palliative procedures begin within the first week of life [277,278,279,280]. Nevertheless, the prognosis is grim, with only two-thirds of children diagnosed with HLHS surviving to 5 years of age and one-third dying before the first surgery [275]. For cases surviving beyond five years, there is an increased risk of heart, digestive, and liver complications, as well as reduced exercise tolerance, and some may even require a heart transplant to survive into adulthood [281].

### 4.4. Avian Models of Environmentally Induced Congenital Heart Defects

Chicken and quail embryos have been extensively used to better understand heart development and the diverse mechanisms that contribute to heart formation [128,282]. Through mechanical or substance interventions, avians allow us to longitudinally study the after-effects of blood flow anomalies and toxins/drugs throughout development (see Table 1). Typically, interventions are performed at the initial stages of development, when the heart is tubular, and show the adaptation of the heart to the insult—from the immediate response to chronic responses and aftereffects [19,283,284]. Unlike mouse or zebrafish models, which are amenable to genetic modifications, avian models are uniquely amenable to interventions that modify blood flow (without altering genetics). Moreover, avian embryos allow easy longitudinal evaluation of cardiac function using optical coherence tomography (OCT) during early tubular heart stages and ultrasound imaging (echocardiography) later during development. Because avian embryos develop in the egg, a simple window in the eggshell is enough to perform in vivo monitoring (OCT and echocardiography) for longitudinal follow-up, with minimum disruption to the developing embryo.

Manipulating blood flow through interventions has enabled detailed studies of the relationship between hemodynamic stresses/stretches and cardiac structure and their role in congenital heart defects. Scientists use avian embryos to recapitulate cardiovascular anomalies that occur in human babies (e.g., VSD, TOF, DORV), enabling investigations of the morphological and molecular mechanisms responsible for cardiac malformations, e.g., [235,245,283,285].

#### 4.4.1. Mechanical Interventions

Mechanical interventions in avian models use tools such as sutures, clips, or lasers to disrupt blood flow in the developing embryo and its heart. These hemodynamic interventions lead to a range of heart malformations seen in human babies with congenital heart disease [18,236,245,283,284,286,287,288,289,290,291,292,293,294].

**Outflow tract banding (OTB):** A suture (band) is placed around the outflow tract portion of the tubular heart and tightened to restrict blood flow out of the heart. The suture can be placed and removed at various times, depending on experimental requirements and desired consequences [283]. While the suture is in place (usually not more than 24 h), normal blood flow is perturbed, resulting in increased blood pressure and elevated peak velocities at the banded region, which leads to increased wall shear stress in the outflow tract [19,295,296]. Along with hemodynamic changes, outflow tract banding (OTB) also leads to structural cardiac changes, including shortening or elongation of the ventricles and a spiraling pattern appearing in the ventricular trabeculae [177,289]. OTB results in isolated VSD, DORV [283], and TOF [235,283].

**Vitelline vein ligation (VVL):** Hemodynamic forces are also altered in avian embryos by placing a clip or suture in one of the two vitelline veins that drain into the tubular heart [173]. Vitelline vein ligation (VVL) blocks flow through one of the two vitelline veins that feed blood to the heart (typically the right vein) and, hence, reduces blood flow through the heart following clipping/ligation [18,173,245,297]. VVL has been used to model a reduction in placental blood flow, which alters hemodynamic stresses in the developing embryo [245,298]. Reduced blood flow decreases wall shear stress on the heart walls and alters the expression of shear-sensitive genes. With reduced blood flow velocity following VVL, the dorsal aorta (downstream of the heart) changes its diameter to maintain a normal wall shear stress while elastin content decreases and collagen increases in its walls [298]. Thus, hemodynamic changes in the embryo mimicking decreased placental blood flow, induce mechanisms that regulate maturation of the aortic ECM, in turn affecting vascular development as well as cardiac formation. VVL, in addition, can lead to pharyngeal arch artery anomalies and VSDs in chicken embryos [235].

**Left atrial ligation (LAL):** A suture is employed to ligate a portion of the left developing heart atrium [297,299]. Left atrial ligation (LAL) causes an immediate decrease in peak blood velocity in the atrioventricular canal, lack of flow in the left atrium, and redirection of flow to the right ventricle [299,300]. Over time, the LAL hemodynamic profile leads to structural heart abnormalities that culminate in HLHS with an underdeveloped left ventricle and single ventricle heart anatomy [290,291,301]. Structural heart abnormalities leading to HLHS in LAL are in response to an imbalance of hemodynamic forces between the two sides of the heart, demonstrating the impact of hemodynamics in morphogenesis [291,299,301,302].

**Laser ablation:** Laser ablation can be used to create targeted cardiac tissue perturbations [141,246,303]. Energy laser pulses can ablate cell populations such as the neural crest or secondary heart field [141,303], resulting in TOF and DORV cardiac defects, or create spatially controlled tissue defects that induce changes in hemodynamics in the avian embryo [246]. When directed to the atrio-ventricular endocardial cushions, these targeted defects resulted in smaller and less-cellularized cushions, reduced ventricular inflow velocity, and increased atrioventricular regurgitation [246].

#### 4.4.2. Substance Interventions

Drugs and toxins can also cause birth defects, including congenital heart malformations [304,305,306,307]. Avian models are frequently employed to test the effects of toxins during development due to their ease of access for performing interventions and monitoring embryonic growth [149,305,308].

**Ethanol exposure:** Fetal alcohol syndrome causes birth defects. Studies using the chicken embryo elucidated malformations that appear specifically in the heart [306,309]. When avian embryos are dosed with ethanol, hemodynamic changes take place while ethanol is in the system [304]. These changes include a dose-dependent increase in heart rate and tachycardiac episodes with depressed contractility, together with decreased embryo viability with increased dosage [304,306]. Ethanol alters normal hemodynamic forces in early embryonic development by increasing retrograde flow to the heart, which leads to altered wall shear stress, including increased oscillatory wall shear stress. Together, hemodynamic changes lead to the underdevelopment of atrio-ventricular cushions and later, during development, result in structural heart defects, including abnormal valves [304,306,309]. Embryos that survived the ethanol dosing showed evidence of structural changes in vessels with instances of hemorrhage, hematoma formations, and hemopericardium [309]. Ethanol exposure resulted in VSDs, smaller atrioventricular valves, malformed aortic valves, and ventricular wall thinning that affected cardiac function [306].

**Cyclopamine exposure:** Cyclopamine is found in corn lilies and is known to cause birth defects [305,308,310,311]. Cyclopamine affects secondary heart field proliferation by disrupting sonic hedgehog signaling [149,308], and dosing early-stage chicken embryos with cyclopamine results in DORV, TOF, VSDs, isolated pulmonary atresia and stenosis, and persistent truncus arteriosus (PTA) [308]. Cyclopamine-intervened embryos also exhibit an abnormal vascular network [312].

**Hyperglycemia exposure:** Maternal diabetes exposes developing babies to increased blood glucose levels or hyperglycemia [313,314] and increases the odds of babies developing congenital heart defects by 5-fold [315,316]. Hyperglycemia in avian embryos leads to delayed development and early changes in heart structure and hemodynamics [317,318].

In summary, the avian embryo model allows longitudinal monitoring of the embryo and its heart to determine how an early environmental or signaling anomaly continues to affect heart formation, leading to heart defects [305,308]. Importantly, avian studies have allowed the discovery of mechano-transduction mechanisms that modulate genetic programs to induce heart defects.

## 5. Cardiac Regeneration

Through evolution and development, the heart has adapted its form and function to satisfy increasingly demanding needs. A remarkable trait is the ability of the heart to self-regenerate, for example, in zebrafish and neonatal mice [319,320]. This ability is lost in the adult human and mouse hearts, as cardiac cells either cease to proliferate or proliferate at extremely low rates.

Efforts are underway to understand how neonatal hearts regenerate and how zebrafish hearts can continue to self-regenerate even in adult stages, with the hopes of one day enabling the self-regeneration of human hearts; see [321,322,323,324]. Moreover, understanding cardiac developmental programs offers the hope of unraveling mechanisms that will allow cardiac cells to proliferate and/or for circulating stem cells to differentiate into cardiac cells and regenerate cardiac tissues [325,326,327]. The last decades have brought tremendous advances in our knowledge, which currently allow us, for instance, to differentiate stem cells into myocardial cells that contract and even self-organize to form cardiac organoids to study heart diseases [328]. Yet the regeneration of cardiac tissues is still elusive. This is in part because of the complexity of the heart structure, which imposes constraints on new cells trying to integrate into existing cardiac tissue. For the heart to regain function, new cells need to be in the right orientation, have the right strength, and properly ‘connect’ to neighboring cells and the extracellular environment. Tissue constructs are being developed to facilitate this integration [325,329]. More research, however, is needed to achieve clinical cardiac regeneration. Independent efforts to understand developmental and evolutionary mechanisms will nevertheless continue to contribute to the development of increasingly sophisticated tools to understand and cure heart disease.

## 6. Concluding Remarks

The heart’s ability to adapt over evolutionary and developmental timescales has allowed for increased interspecies diversity on a global scale, and for increased viability and performance on an individual scale.

Over time, species have evolved to have different and often more complex needs, such as higher metabolic demands to support thermoregulation, the ability to survive in diverse aquatic and terrestrial environments, and the ability to fly and dive. As different species adapted to thrive in their environment, the heart has followed suit and adapted its structure, function, or both to better support the organisms’ needs. In turn, this adaptability required mechanisms that could sense and respond to environmental cues. Without the heart’s ability to adapt to the ever-changing needs across species and across evolution, it is reasonable to speculate that we would not see the same diversity and complexity of species that we see today.

Likewise, during embryonic development, the heart continuously changes its structure to satisfy the changing needs of the growing embryo. In a relatively short time, the human embryonic heart transforms from a primitive tubular structure into a four-chambered heart with valves and fetal shunts that then close after birth, separating the systemic and pulmonary circulations. The mechanics of blood flow play a fundamental role in guiding embryonic heart growth by adapting to diverse conditions to increase survivability. Maladaptation, however, can result in congenital heart disease.

The avian embryo is more amenable than other animal models to blood flow alterations during early development and is thus used to study the complex adaptations in response to altered hemodynamic stresses. This is because the avian embryo is easy to access inside the eggshell. Using mechanical and chemical interventions, researchers have manipulated biophysical cues or signaling pathways, quantifying changes that occur and determining what is required to override such changes. Research using avian embryos has allowed a unique window into what happens during development and how environmental changes, including blood flow anomalies, affect the development of the embryonic heart.

More research is needed to fully understand the genetic and environmental causes of cardiac malformations and how they interact during heart formation. Likewise, more research is needed to understand how the heart tissues respond to diverse environments so that we can better tackle efforts to amend the heart through diverse interventions and regeneration. A better understanding of biophysical stimuli and the mechanisms of common adaptations will improve our ability to predict, manage, and treat individuals who experience pathophysiological heart responses, whether these responses occur during cardiac development or in the fully developed heart. Meanwhile, understanding how organisms and their hearts adapted to live in very different environments and how diverse species developed unique solutions to pump blood could give clinicians and researchers clues on how to best manage heart disease.

## Figures and Tables

**Table 1:** Interventions on chicken embryos that lead to heart defects and associated hemodynamic changes. Pictures created with BioRender.com: https://www.biorender.com/ (accessed on 5 March 2024).

Intervention	Resulting Heart Defect	Hemodynamic Perturbations
Outflow tract banding (OTB) 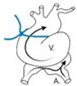	VSD, DORV, TOF. Right and left ventricular remodeling, spiraling pattern in trabeculae, pharyngeal arch artery malformation.	Elevated peak velocities at banded regions, increased blood pressure, higher than normal wall shear stress.
Vitelline-vein ligation (VVL) 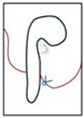	VSD, DORV, pharyngeal arch artery malformation, valve anomalies.	Decreased cardiac blood flow velocity and stroke volume.
Left atrial ligation (LAL) 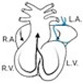	HLHS, single ventricle anatomy, underdeveloped left ventricle, increased right ventricle size (volume).	Immediate decrease in peak blood velocity in atrioventricular canal, significantly decreased flow in the left portion of the heart, flow redirection to right ventricle.
Laser ablation 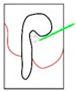	Smaller and less cellularized atrioventricular cushions.	Lower ventricular inflow velocity and atrioventricular regurgitation.
Ethanol 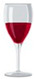	VSD, aortic and atrioventricular valve defects, thinner ventricular walls, increased vascular tortuosity.	Increased heart rate, tachycardiac episodes, and depressed contractility. Increased retrograde flow to the heart and altered wall shear stress.
Cyclopamine 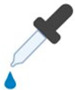	DORV, TOF, PTA, pulmonary atresia and stenosis, increased aortic diameter, irregular vascular network.	Unknown.
Hyperglycemia ^1^ 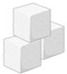	VSD, TOF, PTA, HLHS, aortic coarctation, myocardial hypertrophy.	Decreased stroke volume and contractility due to hypertrophy. Early in development (tubular stages) increased retrograde flow and decrease blood flow rates.

^1^ Data from human babies born from diabetic mothers.

## Data Availability

No new data were created or analyzed in this study.
